# Significant Leukocytosis with Hypereosinophilia Secondary to *Trichuris trichiura* in Adult: A Case Report

**DOI:** 10.3390/clinpract11040094

**Published:** 2021-10-26

**Authors:** Nasturah Abdullah, Noorhida Baharudin, Farah Roslinda Mohd Rustam, Zalizah Khalid

**Affiliations:** 1 Department of Primary Care Medicine, Faculty of Medicine, Universiti Teknologi MARA, Selayang Campus, Batu Caves 68100, Malaysia; nasturahabdullah@gmail.com; 2 Department of Medical Microbiology and Parasitology, Faculty of Medicine, Universiti Teknologi MARA, Sungai Buloh Campus, Sungai Buloh 47000, Malaysia; farah7757@uitm.edu.my; 3 Department of Pathology, Faculty of Medicine, Universiti Teknologi MARA, Sungai Buloh Campus, Sungai Buloh 47000, Malaysia; zalizah@uitm.edu.my

**Keywords:** leukocytosis, hypereosinophilia, adult, *Trichuris trichiura*

## Abstract

Eosinophilic leukocytosis can be attributed to a number of clinical conditions such as parasitic infection, allergies, and neoplasms. Parasitic infection is the main cause of eosinophilia; however, a marked leukocytosis with hypereosinophilia secondary to *Trichuris trichiura* in adults has not been previously reported. We describe a case of a 39-year-old man who presented with fever and diarrhea. The investigation revealed a white blood cell (WBC) count of 20.69 × 10^9^/L with an absolute eosinophil count of 12.44 × 10^9^/L. Fecal microscopic examination demonstrated *T. trichuria* eggs. The WBC count returned to normal following treatment with albendazole. The literature pertaining to hematological findings associated with *Trichuris trichiura* is explored in this report. This case highlights that a significant elevation of leukocyte count with hypereosinophilia can be one of the manifestations of trichuriasis infection in adults. Empirical treatment with anti-helminthic agents may play a role in suspected cases to avoid severe complications, such as Trichuris dysentery syndrome.

## 1. Introduction

Eosinophilic leukocytosis can be caused by a variety of medical conditions, including allergies, infections, tumors, and immune diseases [1]. In countries with little exposure to parasites, allergies and reactions to medications are the main etiologies contributing to eosinophilia [2]. Eosinophilia can be one of the manifestations of the drug reaction with eosinophilia and systemic symptoms (DRESS) syndrome, and the most common offending drugs are the antiepileptic agents, allopurinol, and antibiotics [3]. The DRESS syndrome should be suspected in a patient who receives new medication and develops cutaneous eruption, systemic symptoms (fever, lymphadenopathy), hematological abnormalities (eosinophilia, atypical lymphocytosis), as well as evidence of visceral organ involvements, such as abnormal liver and renal function tests [3].

In general, a parasitic infection is the main cause of eosinophilia, the most common being filariasis, followed by schistosomiasis, hookworm, *Trichuris* spp., and *Strongyloides* spp. [4,5]. Among the parasites, filariasis has a higher degree for causing eosinophilia [4,5]. *Trichuris trichiura*, commonly known as whipworm, rarely leads to hypereosinophilia (eosinophil count ≥ 1.5 × 10^9^/L), especially among adults living in urban areas [6]. We present a case of a 39-year-old man with significant leukocytosis and hypereosinophilia from a *Trichuris trichiura* infection.

## 2. Case Presentation

A 39-year-old man was referred to our clinic with persistent leukocytosis. He complained of feeling feverish with myalgia for one month. He also had diarrhea, up to ten times per day, for five days prior to his presentation to the clinic. There was no abdominal pain or vomiting, and he was able to tolerate orally well. He did not have other constitutional symptoms, such as weight loss or night sweats. He did not have any rashes, cough, shortness of breath, or chest pain. All other systems review was unremarkable. There was no similar episode among other family members.

He worked as an operations manager in a factory and was primarily responsible for overseeing food preparation in the factory dining room. Thus, he was required to touch the raw materials, such as vegetables as part of the quality control measures. He lived in an urban residential area equipped with adequate sanitation and sewerage systems. There was no history of recent travel or recreational activities in the preceding six months. He was an active smoker of 24-pack-years. His past medical history was unremarkable. Specifically, he did not have any history of allergies or atopic medical conditions. He had not been on any medications for the past three months.

Physical examination did not show pallor, rashes, or finger clubbing. He was afebrile with temperature of 36 °C. His heart rate and blood pressure were all within the normal range. Abdominal examination was normal with no hepatosplenomegaly. He did not have any lymphadenopathy. His complete blood count (CBC) showed a white blood cell (WBC) count of 20.69 × 10^9^/L with an absolute eosinophil count (AEC) of 12.44 × 10^9^/L, hemoglobin of 17.1 g/dL, and platelet counts of 365 × 10^9^/L. Hepatic and renal function tests were within the normal range. A peripheral blood film (PBF) showed leukocytosis and eosinophilia with no blast or immature granulocytes seen. There was also polycythemia, and the red bloods cells appeared normocytic and normochromic, with no nucleated red blood cells. The serum erythropoietin (EPO) level was normal (6.4 IU/L).

**Figure 1:**
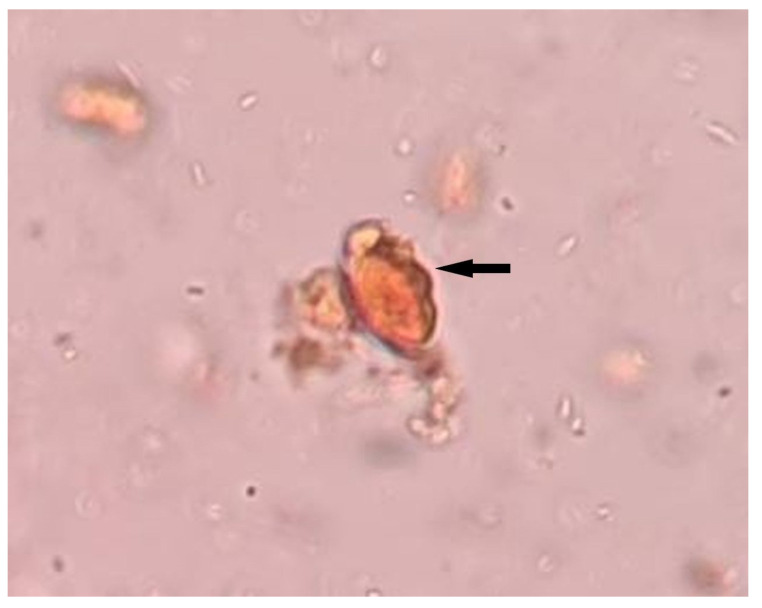
Stool for ova and cyst shows presence of *T. trichiura*’s egg (black arrow).

The fecal microscopic examination showed *T. trichiura*’s egg, characterized by a barrel-shaped, thick-shelled egg with a pair of polar “plugs” at each end (Figure 1). He was treated with albendazole 400 mg tablets twice a day for three days. The resolution of clinical symptoms and normalization of leukocyte counts following treatment supported the diagnosis of hypereosinophilia secondary to trichuriasis. Based on the findings of peripheral blood film (absence of immature granulocytes, no blast cells), normal platelet count, and normal serum EPO level, myeloproliferative neoplasms were unlikely [7]. The elevated hemoglobin was probably due to relative polycythemia (pseudopolycythemia), contributed by his smoking habit, thus an appropriate smoking cessation counselling was offered to the patient. The cytogenetic and molecular testing, such as identification of platelet-derived growth factor receptor alpha gene (*PDGFRA*) and platelet-derived growth factor receptor beta gene (*PDGFRB*) rearrangements, was not carried out due to initial findings of *T. trichiura*’s egg in fecal microscopy and the normalization of eosinophil counts following treatment. His CBC results pre and post treatment with albendazole is shown in Table 1.

Other possible diagnosis such as DRESS colitis was highly unlikely as this patient had not been on any new medications which may trigger DRESS syndrome. Furthermore, apart from hypereosinophilia, he did not have any symptoms and signs suggesting this condition, such as rashes and enlarged lymph nodes. Other laboratory findings including liver and renal function tests were also normal.

## 3. Discussion

Eosinophilia refers to an elevation of absolute eosinophil counts of more than 0.5 × 10^9^/L [8]. Continuing on this spectrum, hypereosinophilia (HE) is defined as a significant elevation of AEC of equal or more than 1.5 × 10^9^/L [2,8]. HE can be subdivided into clonal (or primary), reactive (or secondary), familial, and idiopathic HE [2,8]. The primary form of HE is classified in the context of hematological malignancies [2]. Meanwhile, reactive HE is caused by underlying condition such as infections, allergies, drug reactions, autoimmune disorders, or solid tumors [2]. Idiopathic HE is concluded after the reactive and hematopoietic malignancies are excluded following thorough and comprehensive investigations [8]. If HE occurs in a familial cluster and after the primary or secondary cause has been ruled out, the term familial HE is used [2,8].

Reactive HE can be caused by various medical conditions such as infections, autoimmune diseases, malignancy, drug reactions, allergies, and hypersensitivity [8]. Drug-induced eosinophilia should be suspected among those with a history of recent drug use, although the reactions may be delayed for up to six weeks in some instances [8]. The DRESS syndrome is a more severe manifestation of adverse drug reaction, characterized by eosinophilia, extensive skin rashes, systemic symptoms, and multiple organ involvement [3]. This condition may potentially be life-threatening, and the symptoms are diverse, thus various diagnostic criteria have been proposed to support the diagnosis of DRESS, such as the RegiSCAR criteria [9]. While eosinophilia and diarrhea can be a first manifestation of the DRESS syndrome [10], we have excluded this etiology in our patient due to the lack of other visceral manifestation of DRESS syndrome. Furthermore, our patient did not fulfill the criteria for the diagnosis of this syndrome, which requires at least three of the following findings: (i) fever > 38 °C, (ii) lymphadenopathy at a minimum of two sites, (iii) evidence of involvement of at least one internal organ, and (iv) blood count abnormalities [9]. Other possible etiologies of reactive eosinophilia include solid tumors, such as renal cell carcinoma and breast cancer [8]; thus, a thorough clinical assessment, supported by appropriate investigations as indicated are essential, especially in the case of persistent eosinophilia.

Parasitic infections are the major cause of HE in tropical countries [2]. The most common parasitic infection causing eosinophilia is filariasis, followed by schistosomiasis and hookworm [4,5]. It was found that repetitive and prolong exposure to T-lymphocytes by parasites will lead to eosinophilic activation by the release of interleukin (IL) 3, 4, and 5, resulting in polyclonal eosinophils expansion [6]. Parasites tend to elicit marked eosinophilia during their migratory phase after their product comes into contact with immune cells [2,11]. Degranulation of granulated eosinophils within the tissue, releasing their performed mediators, will lead to tissue dysfunction and damage [2]. There is no reliable and precise level of eosinophilia that can trigger tissue or organ damage, but an arbitrary threshold of eosinophilia >1.5 × 10^9^/L was classically considered as a risk [8]. Intestinal helminth infection is usually assumed as the main cause of eosinophilia among resource-poor populations [2]. A study done in a remote area in Brazil showed that eosinophilia occurred in half of the population and it was significantly associated with the presence of intestinal helminths [5]. Among immigrants from Africa, eosinophilia was frequently observed, with filariasis as the main etiology, followed by schistosomiasis and hookworm [4].

Trichuriasis infection begins with the ingestion of food or water contaminated with embryonated eggs of *T. trichura* [6,12]. The eggs hatch to form larva at the small intestine and then migrate to the cecum, where they mature into adult worms [6,12]. Female worm starts releasing eggs within the feces after a month of infection [6]. Infected individuals are commonly asymptomatic, but heavy colonic infection may cause Trichuris dysentery syndrome (TDS). TDS is mainly seen in children and characterized by mucoid diarrhea, rectal bleeding, rectal prolapse, iron deficiency anemia, and finger clubbing [6,13,14].

In Eastern Europe, *Trichinella* Spiralis is the most common parasites causing eosinophilia [15], while in Asia, *T. trichiura* is the main causative organism [6]. Asia contributes to 67% of global soil-transmitted helminths with the highest incidence in India (21%), followed by China (18%) [16]. Helminth infections are more common in the rural area compared to the urban, most possibly due to socioeconomic status, sanitary measures, and types of water supply [17]. The prevalence of trichuriasis is higher in school-age children living in tropical countries [14]. Compared to adults, *T. trichura* infection is more common in children due to the higher probability of exposure to contaminated soil when playing in the infected area [6]. In adults, infection or reinfection occurs mainly through the consumption of contaminated vegetables [6]. Recent studies have shown that mild to moderate trichuriasis in children can affect growth, physical health, and cognitive abilities [6,12,18].

A significant eosinophilia is not usually observed in trichuriasis, because this parasite usually remains in the colon and the mucosal inflammation is rare [12]. An earlier study among adults infected with intestinal helminths in a rural area in Brazil showed that the leukocyte counts ranged from 3.3 × 10^9^/L to 16.1 × 10^9^/L (median, 7.2 × 10^9^/L) with the eosinophil counts between 0.04 and 1.46 × 10^9^/L (median, 0.455 × 10^9^/L) [5]. This study also concluded that while the probability of eosinophilia and HE increases with the number of parasite species present, the probability of leukocytosis did not [5].

While eosinophilia is commonly seen with trichuriasis infection [2,11], the literature pertaining to HE with marked leukocytosis secondary to this infection in adult remains scarce. Previous cases reported mild eosinophilia with normal leukocyte counts in adult with trichuriasis [19,20]. A case series from Korea reported that among four adult cases of trichuriasis, only one showed mild eosinophilia (AEC 0.48 × 10^9^/L) with normal leukocyte counts [19]. Another case in Korea also reported normal leukocyte counts of 5.7 × 10^9^/L without eosinophilia in a young adult with TDS [20]. Much higher leukocyte and eosinophil counts were previously reported in children infected by *T. trichiura* [21,22,23]. In one pediatric case of TDS in India, leukocytosis and HE were present, with leukocyte counts of 80 × 10^9^/L and AEC of 40 × 10^9^/L [23]. Another case study from Malaysia also reported that among four children diagnosed with TDS, only two had leukocytosis and HE, with the highest leukocyte counts of 34.94 × 10^9^/L and the AEC of 22.78 × 10^9^/ L [24].

In summary, a significant elevation of leukocyte count with hypereosinophilia can be one of the manifestations of trichuriasis infection in adults. Empirical treatment with anti-helminthic agents should be considered in suspected cases, especially in resource-poor populations where this infection is more prevalent.

## Figures and Tables

**Table 1:** Complete blood counts with differentials before and after treatment.

Parameters	Pre-Treatment with Oral Albendazole	Post-Treatment with Oral Albendazole	Reference Range
Hb (g/dL)	**17.1**	**18.3**	13.0–17.0
HCT (%)	49.1	**52.8**	40.0–50.0
Platelet (×10^9^/L)	365	328	150–410
WBC (×10^9^/L)	**20.69**	9.58	4.00–10.00
WBC differentials (%)			
Neutrophils (%)	12.6	48.2	40.0–80.0
Lymphocytes (%)	22.0	39.1	20.0–40.0
Monocytes (%)	5.0	7.5	2.0–10.0
Eosinophils (%)	**60.1 (AEC: 12.44 × 10^9^/L)**	4.6	1.0–6.0
Basophils (%)	0.3	2.6	0.0–2.0

AEC: Absolute eosinophil count.

## Data Availability

Not applicable.
